# Association Between Plasma Metabolites and Psychometric Scores Among Children With Developmental Disabilities: Investigating Sex-Differences

**DOI:** 10.3389/fpsyt.2020.579538

**Published:** 2020-12-22

**Authors:** Jennie Sotelo-Orozco, Leonard Abbeduto, Irva Hertz-Picciotto, Carolyn M. Slupsky

**Affiliations:** ^1^Department of Public Health Sciences, University of California, Davis, Davis, CA, United States; ^2^Department of Psychiatry and Behavioral Sciences, University of California Davis Health, Sacramento, CA, United States; ^3^MIND Institute, University of California Davis, Sacramento, CA, United States; ^4^Department of Nutrition, University of California, Davis, Davis, CA, United States; ^5^Department of Food Science and Technology, University of California, Davis, Davis, CA, United States

**Keywords:** autism spectrum disorder, down syndrome, adaptive behavior, cognitive scores, maladaptive behavior, sex-differences, metabolites, metabolomics

## Abstract

**Background:** Developmental disabilities are defined by delays in learning, language, and behavior, yet growing evidence has revealed disturbances in metabolic systems that may also be present. Little is known about whether these metabolic issues contribute to the symptoms or severity of these disabilities, or whether sex plays a role in these associations, given that boys are disproportionately affected by some developmental disabilities. Here we sought to investigate the correlation between psychometric scores, sex, and the plasma metabolome.

**Methods:** The plasma metabolomes of children with autism spectrum disorder (ASD; *n* = 167), idiopathic developmental delay (i-DD; *n* = 51), Down syndrome (DS; *n* = 31), and typically developing controls (TD; *n* = 193) were investigated using NMR spectroscopy. Spearman rank correlations and multiple linear regression models (adjusted for child's neurodevelopmental diagnosis, child's sex, child's age, child's race/ethnicity, maternal age at child's birth, and parental homeownership) were used to examine the association between plasma metabolites and sex in relation to psychometric measures of cognitive skills, adaptive behavior, and maladaptive behavior in our study population.

**Results:** Higher levels of metabolites involved in cellular energy and mitochondrial function among children with ASD (fumarate and cis-aconitate), DS (lactate), and TD (pyruvate) are associated with poorer cognitive and adaptive subscales. Similarly, higher o-acetylcarnitine associated with deficits in cognitive subscales among all DS cases and TD boys, and carnitine correlated with increased maladaptive behavior among girls with ASD and girls with DS. Among children with DS, elevated myo-inositol, ornithine, and creatine correlated with poorer scores across several subscales. Even among TD cases, elevated 3-hydroxybutyrate correlated with decreased receptive language. In contrast, higher levels of glutamate were associated with better socialization skills among ASD cases. Even after adjusting for the child's neurodevelopmental diagnosis, sex, and other possible confounders, key metabolites including glycolysis metabolites (lactate and pyruvate), ketone bodies (3-hydroxybutyrate and acetoacetate), TCA cycle metabolites (cis-aconitate and fumarate), as well as ornithine were associated with deficits in multiple domains of cognitive function, adaptive skills, and aberrant behaviors.

**Conclusions:** Our results highlight that some plasma metabolites may relate to specific functional subdomains within cognitive, adaptive, and behavioral development with some variation by diagnosis and sex.

## Introduction

In the United States, it is estimated that nearly 17% of children between the ages of 3 and 17 years have one or more developmental disabilities (1). Some common developmental disabilities include Down syndrome (DS), fragile X syndrome, autism spectrum disorder (ASD), cerebral palsy, and intellectual disability. Although these disorders specifically affect learning, language, and behavior, these conditions can also be accompanied by disturbances in metabolism (2). Numerous studies have shown metabolic and systemic imbalances in individuals with ASD, including increased oxidative stress (3, 4), decreased methylation capacity (4–6), impaired sulfur metabolism (7, 8), gut microbiome dysbiosis (9–14), and dysregulated energy metabolism (15–17). Altered metabolism has also long been identified in DS as a consequence of overexpression of genes/proteins encoded on chromosome 21 (such as superoxide dismutase 1) and contributing to the underlying oxidative stress in DS (18, 19). Gene products of chromosome 21 also interact with other genes or proteins on other chromosomes resulting in broad metabolic consequences (20). Our previous study investigated metabolic alterations in a subset of CHARGE (Childhood Autism Risks from Genetics and the Environment) Study participants with ASD, DS, and idiopathic developmental disabilities (i-DD) compared to typically developing children (17). Disturbances in mitochondrial dysfunction, urea cycle, and amino acid/nitrogen metabolism were found among children with ASD, whereas children with DS had alterations in lipid metabolism. Similarities and differences between conditions were also observed, however, with perturbed one-carbon metabolism pathways among children with DS and ASD—yet within one-carbon metabolism, the folic acid-folate cycle was affected in ASD cases, whereas the methionine cycle was affected in DS cases. Similarities were also observed among children with DS and i-DD in the energy-related tricarboxylic acid (TCA) cycle. However, whether these metabolic abnormalities correlate with specific behavioral phenotypes and developmental scores has yet to be adequately investigated—the present study is a continuation of our previous work to attempt to address this specific issue.

A few studies have suggested that metabolic alterations may be contributing to the severity of the ASD phenotype. A small study on twenty Egyptian children ages 2–7 years old found that the severity of autism symptoms, as measured by the Childhood Autism Rating Scales (21) (CARS), inversely correlated with blood levels of some essential amino acids. The higher the CARS scores, the lower the blood levels of leucine, isoleucine, phenylalanine, methionine, cysteine, serine, and tyrosine (22). Others have also found that decreased levels of phenylalanine correlated with more severe autistic features as measured by the Severity of Autism Scale (SAS) (23). Indeed, altered metabolite profiles may have consequences for behavior: for example, decreased blood amino acid levels of tryptophan and phenylalanine may lead to lower concentrations of neurotransmitters given their function as precursors. These metabolic perturbations may also contribute more broadly to the symptoms or severity of developmental delay (2). Therefore, the present study aimed to investigate these complex interactions by measuring how biomarkers in the blood (products and byproducts of metabolic pathways) correlate with developmental/behavioral scores among children with ASD, DS, i-DD, and TD and to examine whether these associations affect boys and girls differently. The literature establishes that ASD affects ~4 times more males than females (24, 25), and 1.3 more males than females are born with DS (26). Hence, we also sought to determine whether the associations between metabolic markers and developmental/behavioral measures differ between boys and girls.

## Methods

### Study Population

All children in the present study are a subset of the Childhood Autism Risk from Genetics and Environment (CHARGE) Study (27). The CHARGE study is a population-based case-control study which aims to uncover the environmental causes of ASD and examine genetic factors and the interactions between genes and environment in the etiology of ASD. The CHARGE study is a large ethnically diverse population. Details about the study have previously been published (27). Eligible children met the following criteria: (a) aged 24–60 months at recruitment, (b) living with a biological parent who speaks English or Spanish, (c) born in California, and (d) residing in the study catchment. The study catchment area included regions throughout the state of California, however, most participants were from the greater Sacramento area in Northern California. CHARGE Participants were sampled from three strata: children with ASD, children with a developmental delay but not ASD (DD), and children from the general population controls. ASD and DD children were recruited from the State of California Department of Developmental Services (DDS). The primary aim of CHARGE was to investigate ASD, and, therefore, controls were matched for frequencies on age, sex, and broad geographic distribution of the autism cases. After thorough clinical testing at the UC Davis MIND Institute (Sacramento, CA), each child was assigned their diagnosis (ASD, DD, or typical development, TD).

**Figure 1:**
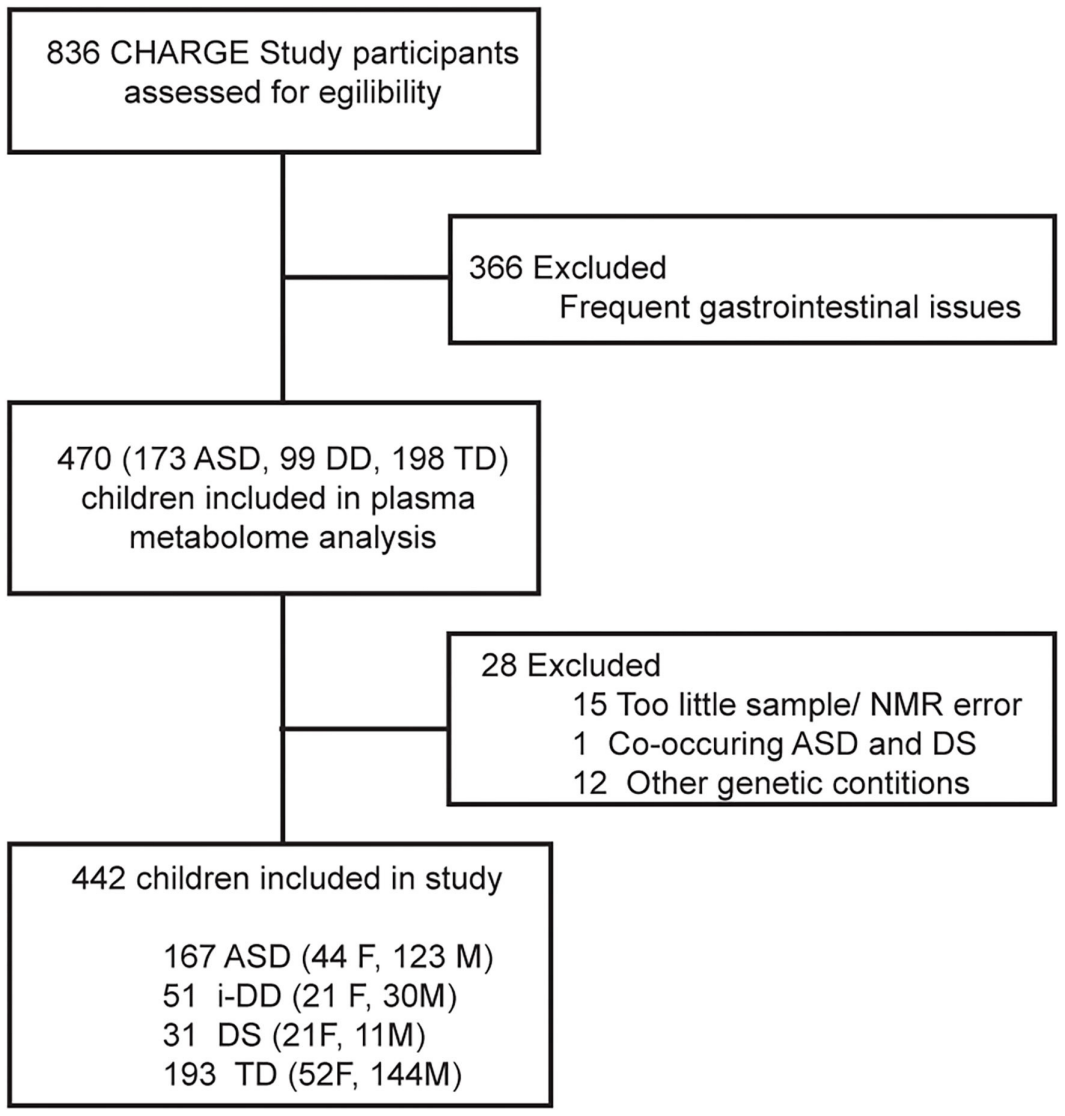
Flow chart of the study population.

A total of 836 children from CHARGE were considered for this analysis who had sufficient plasma for metabolomics analysis. Individuals with any frequent gastrointestinal symptoms according to parental reports in the last 3 months were excluded (*n* = 366) from further analysis to avoid a potential influence of dysregulated GI function on metabolism. Children with other genetic or metabolic conditions (as specified on the medical history questionnaire) (*n* = 12), too little volume or sample processing errors (*n* = 15), and co-occurring conditions (*n* = 1) were also excluded. Furthermore, the original DD group in CHARGE was subdivided into an idiopathic DD (i-DD) group (with an unknown etiology), and a Down syndrome (DS) group (which was reported by a parent). Our final sample size was composed of a total of 442 children (167 ASD, 51 i-DD, 31 DS, and 193 TD) (Figure 1).

The CHARGE study was approved by the State of California Department of Developmental Services and the institutional review boards at the University of California, Davis, and Los Angeles. Informed consent was obtained before participation and any collection of data.

### Child Neurodevelopmental Assessment and Measures

#### Mullen Scale of Early Learning

All children were administered the Mullen Scale of Early Learning (MSEL), a test of cognitive skills (28). The MSEL is a standardized developmental test for children from birth to 68 months of age. Four sub-scales of the MSEL were administered: visual reception, fine motor, receptive language, and expressive language. The visual reception scale focuses on visual perceptual ability and non-verbal skills (e.g., localizing a target, visual tracking); the fine motor scale involves bilateral and unilateral manipulation (e.g., turning pages in a book, stacking blocks); the receptive language scale provides an assessment of a child's ability to derive linguistic meaning from spoken language, and the expressive language scale assesses a child's ability to use oral language to express meaning. Composite scores were also determined. To avoid floor-effects, all MSEL scores are presented as the developmental quotient (DQ), meaning the ratio of the age equivalent score to the chronological age.

#### Vineland Adaptive Behavior Scales

Adaptive function was evaluated in all children by parent interview using the Vineland Adaptive Behaviors Scales (VABS) 1st edition (29), which covers domains of socialization, daily living skills, motor skills, and communication. The socialization domain encompasses functioning in interpersonal relationships, play and leisure time, and coping skills; daily living skills includes items like feeding self with a spoon or putting things away when asked to do so; motor skills domain involves gross motor items and fine motor items; communication domain covers both expressive language and receptive language. Composite scores were also determined. To avoid floor-effects, VABS scores are also presented as the developmental quotient (DQ), meaning the age equivalent score to the chronological age at the time of testing.

#### Aberrant Behavior Checklist

Parents self-administered the Aberrant Behavior Checklist (ABC) (30) to rate inappropriate and maladaptive behaviors. The ABC is one of the most widely used behavior rating scales and consists of 58 items, each scored on a four-point scale ranging from 0 (=not a problem) to 3 (=problem is severe in degree). The items are assigned to one of five subscales: the first included irritability, agitation, crying (15 items); the second was lethargy and social withdrawal (16 items); the third, stereotypic behavior (seven items); fourth, hyperactivity, or non-compliance (16 items); and fifth, inappropriate speech (four items). Unlike the MSEL and VABS (where higher scores show improvements), higher ABC scores indicate more maladaptive/problematic behavior.

### ^1^H NMR Metabolomics Analysis

Whole blood from each child was collected in a yellow top (Acid Citrate Dextrose) tubes (BD Biosciences, San Jose CA) at the time of the child's enrollment in the study. Plasma was isolated and frozen at −80°C until further analysis. For metabolomics analysis, thawed samples were filtered through an Amicon 3,000 MW cut-off Centrifugal Device to remove lipids and proteins. The water-soluble filtrate was collected, or volume was adjusted with Type I ultrapure water from a Millipore Synergy UV system (Millipore, Billerica, MI) if an insufficient sample was collected. An internal standard (ISTD) containing DSS-D6 ([3-(trimethylsilyl)-1-propanesulfonic acid-d6], 0.2% NaN_3_, in 99.8% D_2_O) was added, and the pH of each sample was adjusted to 6.8 ± 0.1 by adding small amounts of NaOH or HCl. Volumes of HCl and NaOH added were recorded. An aliquot of the mixture was transferred to a labeled 3 mm Bruker NMR tube and stored at 4°C until NMR acquisition (within 24 h of sample preparation).

Samples were run on a Bruker AVANCE 600 MHz NMR spectrometer equipped with a SampleJet autosampler using the NOESY-pre-saturation pulse sequence (noesypr). NMR spectra were acquired at 25°C, with water saturation of 2.5 s during the prescan delay, a mixing time of 100 ms, 12 ppm sweep width, an acquisition time of 2.5 s, eight dummy scans, and 32 transients. All spectra were zero-filled to 128K data points and Fourier transformed with a 0.5-Hz line broadening applied. Spectra were manually phased and baseline-corrected and metabolites were identified and quantified using NMR Suite v8.1 (Chenomx Inc., Edmonton, Canada). The Chenomx profiler is linked to a database containing 339 NMR spectral metabolite signatures valid at a pH between 4 and 9. To ensure metabolite cluster fits remained within the valid Chenomx profiler pH, the average pH was collected [(adjusted pH–pH post-NMR)/2] and manually input into the software before metabolite quantification which was based on the known concentration of the added internal standard. All compounds in the database have been verified against known concentrations of reference NMR spectra of the pure compounds and have been shown to be reproducible and accurate (31, 32). Investigators were blinded to child diagnosis during sample preparation as well as NMR data acquisition and spectral analysis.

### Statistical Analysis

The demographic characteristics of groups were evaluated using the chi-square test for categorical variables and the Kruskal–Wallis test for continuous variables. Shapiro Wilks test was used to test for normality. Two-way ANOVA was used to compare psychometric subscales and metabolite concentrations across diagnosis (Dx) and sex, and to test for an interaction effect (Dx^*^ sex). A total of 59 metabolites of diverse chemical classes were identified in plasma samples. However, metabolites identified in samples but originating from sample preparation (e.g., glucose, citrate, ethanol, and glycerol), or falling below the detection limit for at least 20% of samples (e.g., fructose, maltose, beta-alanine, propionate, mannose, and isopropanol) were excluded in the final analysis. Therefore, a total of 49 plasma metabolites were analyzed in this study, including those involved in amino acid metabolism, glutathione metabolism, glycolysis, homocysteine metabolism, ketone body synthesis, lipid metabolism, TCA cycle, urea cycle, and others (Supplementary Table 1). Metabolite concentrations (μM) were adjusted for any dilutions and log-transformed because of the wide variation and skewed distributions.

For all analyses, the inverse of the ABC scores was used so that similarly to MSEL and VABS, negative regression coefficients for all psychometric outcome scores would indicate poorer performance. Correlations between the plasma metabolites and psychometric scores were computed using Spearman rank correlation coefficients (r). A coefficient |0.0 to 0.19| indicates very weak correlation, |0.2 to 0.39| = weak correlation, |0.4 to 0.59| = moderate correlation, |0.6 to 0.79| = strong correlation, and |0.8 to 1.0| = very strong correlation (33). A heatmap correlation plot was generated on the Spearman rank correlations using R (package “corrplot”). To account for multiple testing, *p*-values from Spearman correlation analysis were adjusted by controlling the false discovery rate (FDR) at 5% using the Benjamini–Hochberg procedure (p.adjust.method = “BH”). Additionally, multiple linear regression (MLR) (glm function) was performed to assess the association between plasma metabolites (independent variable) and psychometric subscales (dependent variable) when controlling for possible confounders. Possible confounders were explored through a directed acyclic graph (DAG) prior to model building. Covariates considered in our DAG were child's sex, child's age at blood draw, child's race/ethnicity, child's year of birth, maternal age at child's birth, maternal race/ethnicity, maternal birthplace, year of blood collection, parental homeownership, and maternal level of education. From the DAG, we then identified a sufficient set of adjustment factors that would remove confounding to minimize bias in the estimated associations between psychometric scores and metabolites in our final models. These included child's neurodevelopmental diagnosis [categorical: ASD, i-DD, DS, and TD (reference)], child sex [categorical: female/male (reference)], child's age at blood draw (continuous) child's race/ethnicity [Hispanic, other, white non-Hispanic (reference)], maternal age at child's birth (continuous), and parental homeownership [no/yes (reference)]. Statistical analyses were performed using R 3.3.3 (R Foundation for statistical computing, version 3.0.1, Vienna Austria. URL http://toexaminethemetaboliR-project.org/) and SAS software version 9.4 (SAS Institute Inc.). KEGG (Kyoto Encyclopedia of Genes and Genomes) pathway database (http://www.genome.ad.jp/kegg/), HMDB (Human metabolome database) (34) (www.hmdb.ca), and MetaboAnalyst Web application (www.metaboanalyst.ca) were used to examine the metabolic pathways.

## Results

### Demographics

Table 1 shows a summary of the study participant demographics. Children with ASD tended to be socio-demographically similar to TD children. I-DD and particularly DS children were more likely to be female than were TD children. This was because the TD sex distribution was matched to the projected ASD sex ratio of 4:1 (males: females), but the DD group was not matched. I-DD cases were more likely to be Hispanic, their mothers tended to be the least educated and their parents least likely to be homeowners, whereas DS and TD parents were the most likely to own their homes. The majority of mothers were born in the U.S. Additionally, as expected, mothers of children with DS tended to be older.

**Table 1:** The study population demographics.

	**ASD**	**i-DD**	**DS**	**TD**	***p*-value^a^**
*N*	167	51	31	193	
Child's sex = Males *n* (%)	123 (73.7)	30 (58.8)	11 (35.5)	141 (73.1)	<0.001
Child's age (months) at blood draw [mean (SD)]	43.96 (10.23)	46.67 (7.88)	44.42 (9.18)	43.09 (9.76)	0.162
Child's race/ethnicity *n* (%)					0.230
White	75 (44.9)	20 (39.2)	15 (48.4)	101 (52.3)	
Hispanic	61 (36.5)	22 (43.1)	12 (38.7)	51 (26.4)	
Other:					
Asian	9 (5.39)	0 (0)	1 (3.23)	6 (3.11)	
Black	6 (3.59)	7 (13.73)	0 (0)	5 (2.59)	
Multi-racial	16 (9.58)	2 (3.92)	3 (9.68)	30 (15.54)	
ADOS-2 comparison score^b^ [mean (SD)]	7.40 (1.44)				
Score: 4–6 *n* (%)	54 (32.3)				
Score: 7–10 *n* (%)	113 (67.7)				
Maternal age (years) at child's birth [mean (SD)]	30.60 (5.50)	29.43 (5.89)	33.77 (6.99)	30.47 (5.60)	0.010
Maximum maternal education *n* (%)					0.373
Bachelor's degree	57 (34.1)	13 (25.5)	13 (41.9)	75 (38.9)	
Graduate or professional	20 (12.0)	3 (5.9)	3 (9.7)	27 (14.0)	
High school/GED or less	31 (18.6)	9 (17.6)	5 (16.1)	28 (14.5)	
Some college	59 (35.3)	26 (51.0)	10 (32.3)	63 (32.6)	
Maternal birthplace *n* (%)					0.247
Mexico	16 (9.6)	4 (7.8)	3 (9.7)	7 (3.6)	
Other	26 (15.6)	7 (13.7)	3 (9.7)	22 (11.4)	
USA	125 (74.9)	40 (78.4)	25 (80.6)	164 (85.0)	
Parental homeownership=Yes *n* (%)	106 (63.5)	31 (60.8)	22 (71.0)	143 (74.1)	0.101

a *Chi-square test used for categorical variables, Kruskal–Wallis Test used for continuous variables*.

b *Autism Diagnostic Observation Schedule 2nd Edition Comparison Score. Comparison score can range from 0 to 10, with 10 being most severe. Only available for ASD cases*.

### Psychometric Scores

We found that children with ASD, i-DD, and DS had significant impairments on all subscales of the MSEL and VABS compared to TD controls (Table 2). Females with ASD, DS, and TD scored significantly higher on all MSEL subscales compared to their male counterparts with the same diagnosis. In contrast, males with i-DD scored significantly higher on all MSEL subscales compared to females with i-DD and compared with both sexes in the DS group. Similar sex-differences were observed for adaptive behavior on the VABS. Females scored significantly higher on all VABS subdomains, except motor skills, where boys tended to score higher.

**Table 2:** Mean (SE) MSEL, VABS, and ABC scores.

	**ASD F** **(*n* = 44)**	**ASD M** **(*n* = 123)**	**i-DD F** **(*n* = 21)**	**i-DD M** **(*n* = 30)**	**DS F** **(*n* = 20)**	**DS M** **(*n* = 11)**	**TD F** **(*n* = 52)**	**TD M** **(*n* = 141)**	**Dx^b^**	**Sex^b^**	Dx ^*^ sex^b^
MSEL^a^											
Visual reception	78.15 (3.53)	68.16 (2.06)	59.19 (4.43)	72.59 (4.57)	57.83 (2.97)	53.41 (3.50)	118.43 (2.76)	110.46 (1.39)	***p*** **<** **0.001**	0.400	**0.004**
Fine motor	72.39 (2.86)	70.02 (1.67)	55.48 (3.88)	67.02 (3.17)	56.44 (2.36)	51.37 (2.14)	110.86 (2.18)	102.79 (1.28)	***p*** **<** **0.001**	0.652	**0.004**
Receptive language	64.21 (4.38)	54.08 (2.42)	52.34 (4.50)	60.94 (3.46)	53.46 (2.84)	47.11 (4.43)	108.23 (2.26)	104.31 (1.47)	***p*** **<** **0.001**	0.305	0.082
Expressive language	61.62 (3.43)	54.94 (2.23)	46.75 (5.00)	59.00 (4.04)	46.94 (2.83)	42.83 (4.13)	108.58 (2.51)	101.96 (1.55)	***p*** **<** **0.001**	0.641	0.037
Composite Score	69.09 (3.11)	61.8 (1.88)	53.44 (4.11)	64.89 (3.35)	53.66 (2.27)	48.68 (3.02)	111.52 (1.90)	104.88 (1.11)	***p*** **<** **0.001**	0.405	**0.006**
VABS^a^											
Communication	56.15 (3.71)	51.61 (2.02)	47.04 (4.09)	57.11 (3.71)	47.64 (2.56)	42.75 (3.83)	113.71 (3.41)	102 (1.57)	***p*** **<** **0.001**	0.317	**0.017**
Daily living	60.27 (2.62)	56.06 (1.23)	51.49 (3.94)	60.76 (3.81)	55.72 (3.08)	53.18 (3.63)	106.12 (2.9)	94.14 (1.67)	***p*** **<** **0.001**	0.314	**0.003**
Socialization	52.37 (3.31)	46.68 (1.65)	56.55 (4.49)	62.00 (4.37)	66.84 (4.73)	53.31 (4.8)	116.76 (3.41)	101.29 (1.73)	***p*** **<** **0.001**	**0.008**	**0.013**
Motor	74.84 (3.7)	76.26 (1.77)	55.69 (4.54)	64.16 (3.51)	52.99 (2.12)	56.39 (4.36)	112.51 (3.26)	103.59 (1.55)	***p*** **<** **0.001**	0.678	**0.024**
Composite score	61.13 (2.76)	57.93 (1.34)	53.00 (3.76)	61.32 (3.30)	55.9 (2.48)	51.9 (3.37)	112.3 (2.62)	100.19 (1.23)	***p*** **<** **0.001**	0.189	***p*** **<** **0.001**
ABC											
Irritability, agitation, crying	13.05 (1.23)	10.43 (0.75)	10.00 (2.33)	6.90 (1.60)	2.20 (0.84)	2.64 (1.06)	3.12 (0.56)	3.03 (0.32)	***p*** **<** **0.001**	0.125	0.269
Lethargy, social withdrawal	8.52 (1.09)	9.05 (0.53)	3.62 (1.05)	2.77 (0.85)	0.85 (0.51)	0.45 (0.16)	0.19 (0.07)	0.48 (0.15)	***p*** **<** **0.001**	0.850	0.787
Stereotypic behavior	4.05 (0.67)	4.33 (0.34)	1.52 (0.72)	2.30 (0.71)	0.35 (0.18)	0.91 (0.46)	0.06 (0.04)	0.06 (0.03)	***p*** **<** **0.001**	0.265	0.839
Hyperactivity, non-compliance	17.02 (1.57)	16.06 (0.94)	13.14 (2.07)	11.83 (2.36)	3.50 (1.13)	4.55 (1.53)	2.13 (0.41)	3.59 (0.44)	***p*** **<** **0.001**	0.958	0.568
Inappropriate speech	4.09 (0.43)	2.76 (0.25)	1.90 (0.53)	1.30 (0.28)	0.05 (0.05)	0.45 (0.28)	0.37 (0.1)	0.30 (0.08)	***p*** **<** **0.001**	0.119	**0.024**

a *Age-adjusted DQ score used*.

b Two-way ANOVA. Only significant associations shown (*p < 0.05. **p < 0.01. ***p < 0.001).

*Higher scores on the MSEL and VABS indicate better performance. In contrast, ABC measures maladaptive behaviors, therefore higher scores on the ABC subscales indicate increased problematic behavior. Two-way ANOVA with main effects and interaction for diagnosis (Dx) and sex. Significant p-values are shown in bold*.

Children with ASD and i-DD had the highest maladaptive scores on the ABC compared to TD controls, although children with ASD consistently had slightly worse scores than children with i-DD. Significant sex by diagnosis interaction was observed for inappropriate speech on the ABC, with ASD girls having the greatest difficulties. Children with DS had better (i.e., lower) ABC scores compared to ASD and i-DD cases, were more comparable to TD children, and even tended to have less irritability, agitation, and crying than TD controls, comparable to other studies (34).

### Correlation Analysis Between Psychometric Scores and Plasma Metabolites

**Figure 2:**
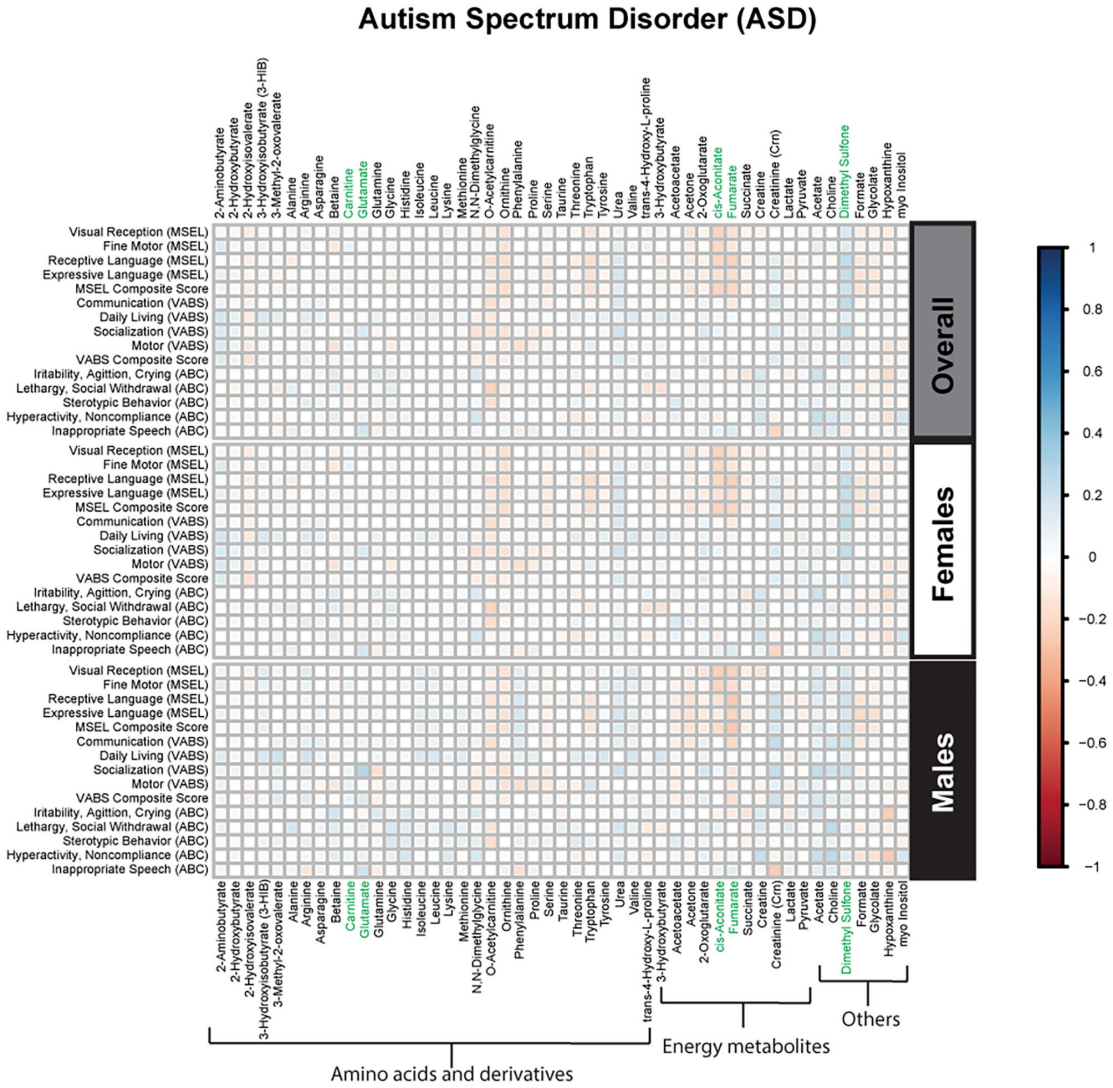
Spearman correlation heatmap of psychometric scores and plasma metabolites for children with Autism Spectrum disorder (ASD) overall and stratified by sex. Positive associations (blue) identify metabolites where higher plasma concentrations correlated with improvement in psychometric scores, while negative associations (red) identify metabolites where higher plasma concentrations correlated with poorer neurodevelopmental or behavioral scores. For correlation analysis, the inverse scores of ABC subscales were used for ease of comparison with other MSEL and VABS scores. Metabolites that remained significant after FDR correction are shown in green.

**Figure 3:**
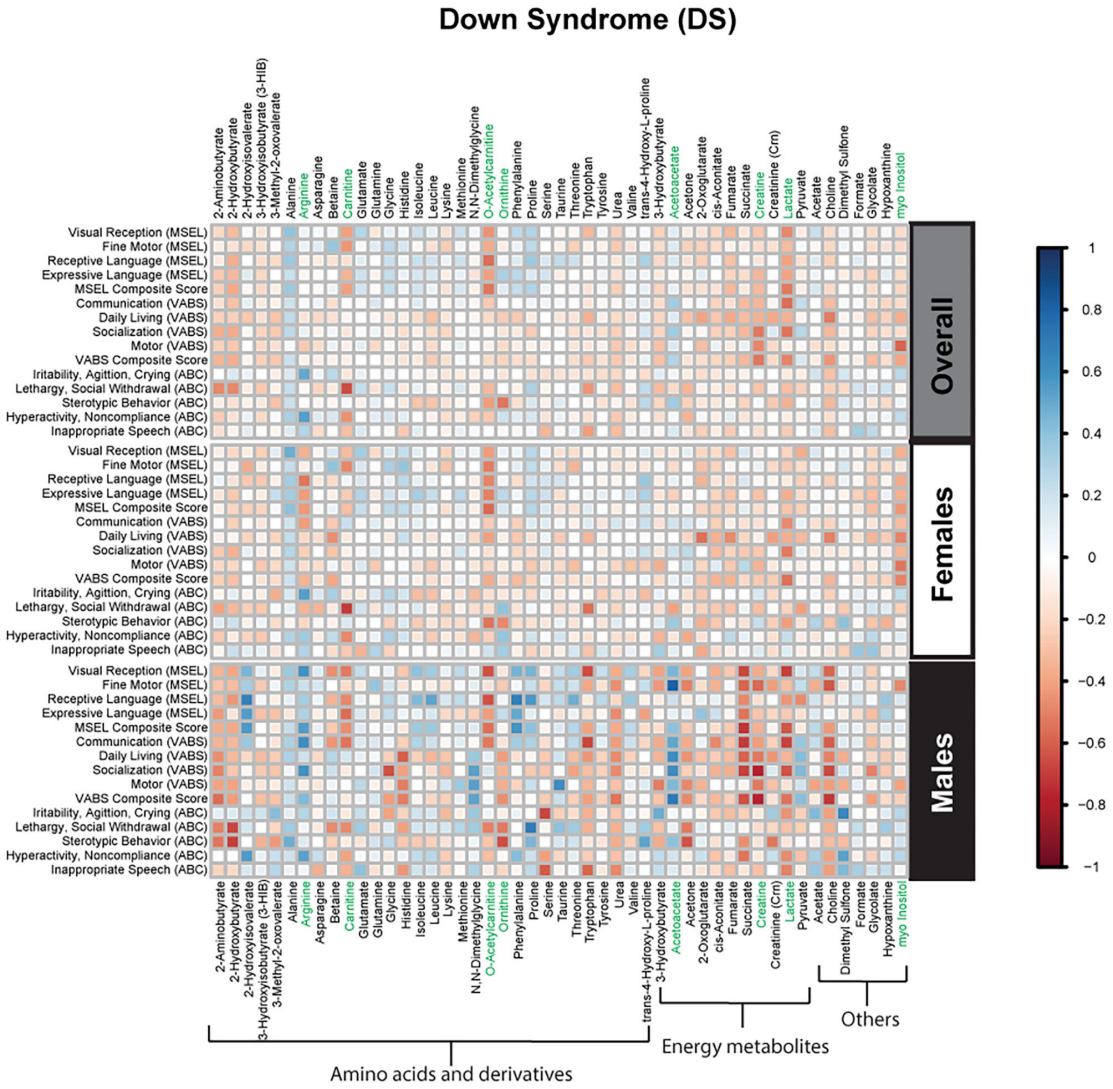
Spearman correlation heatmap of psychometric scores and plasma metabolites for children with Down syndrome (DS) overall and stratified by sex. Positive associations (blue) identify metabolites where higher plasma concentrations correlated with improvement in psychometric scores, while negative associations (red) identify metabolites where higher plasma concentrations correlated with poorer neurodevelopmental or behavioral scores. For correlation analysis, the inverse scores of ABC subscales were used for ease of comparison with other MSEL and VABS scores. Metabolites that remained significant after FDR correction are shown in green.

**Figure 4:**
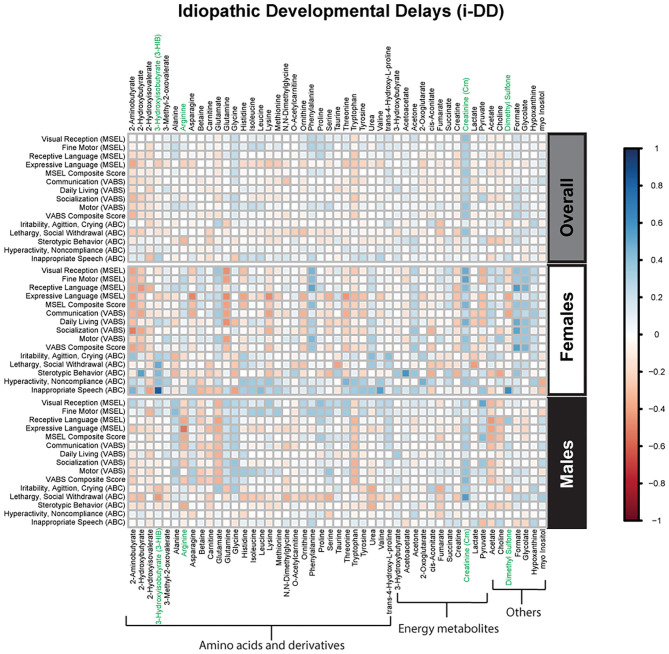
Spearman correlation heatmap of psychometric scores and plasma metabolites for children with idiopathic Developmental Delays (i-DD) overall and stratified by sex. Positive associations (blue) identify metabolites where higher plasma concentrations correlated with improvement in psychometric scores, while negative associations (red) identify metabolites where higher plasma concentrations correlated with poorer neurodevelopmental or behavioral scores. For correlation analysis, the inverse scores of ABC subscales were used for ease of comparison with other MSEL and VABS scores. Metabolites that remained significant after FDR correction are shown in green.

**Figure 5:**
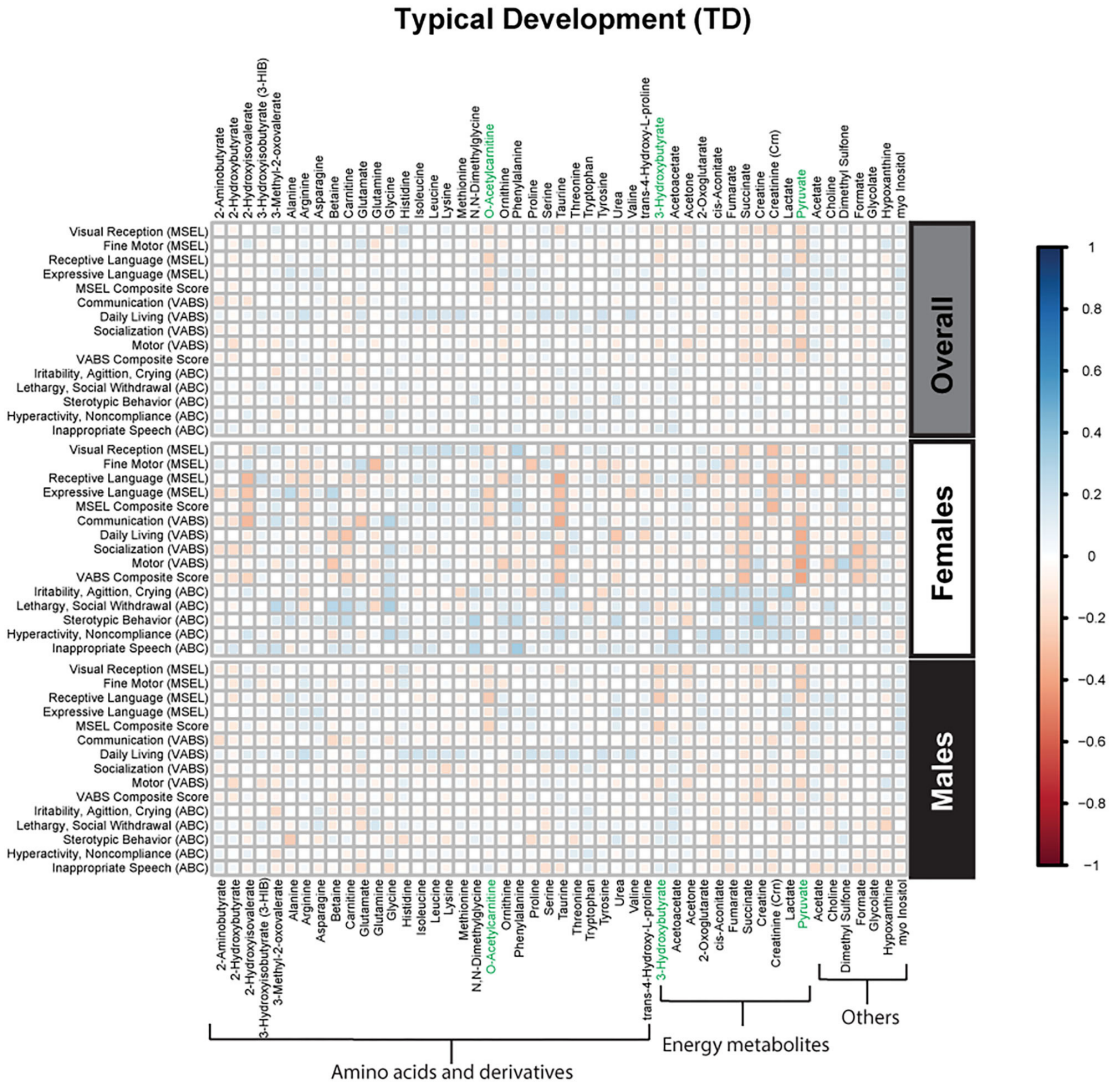
Spearman correlation heatmap of psychometric scores and plasma metabolites for children with typical development (TD) overall and stratified by sex. Positive associations (blue) identify metabolites where higher plasma concentrations correlated with improvement in psychometric scores, while negative associations (red) identify metabolites where higher plasma concentrations correlated with poorer neurodevelopmental or behavioral scores. For correlation analysis, the inverse scores of ABC subscales were used for ease of comparison with other MSEL and VABS scores. Metabolites that remained significant after FDR correction are shown in green.

Spearman correlations were used to explore the association between psychometric scores based on plasma metabolites stratified by diagnosis and sex. We found strong associations between plasma metabolites and cognitive scores among DS cases. Most of these associations were negative (i.e., elevated plasma metabolites correlated with poorer psychometric scores), except acetoacetate which had a positive association with scores. Certainly, an extra copy of chromosome 21 would be expected to interact with genes or proteins on other chromosomes resulting in wide-ranging metabolic disruptions observed. In contrast, predominantly weak correlations (with a mix of positive and negative associations) were found between plasma metabolites and scores among ASD cases. Considering ASD is truly a spectrum with a diversity of symptoms, severity, and abilities, these results are not surprising. However, this may suggest that for ASD, unlike DS, the genetic contributions to metabolism are minimal, and other factors, such as environmental exposures, could have a greater contribution to these metabolic insults. Interestingly, among i-DD children, we found mostly positive correlations (i.e., elevated plasma levels correlated with improved scores) ranging from weak to strong correlations. Even among our control children with typical development, we found some negative correlations, although these were mostly weak. The results of the Spearman correlations across the diagnoses and stratified by sex are presented in Figures 2–5, while metabolites that remained significant after adjusting for multiple comparisons (FDR *p* < 0.05) are summarized in Table 3.

**Table 3:** Spearman rank correlation (r) between plasma metabolites and psychometric scores for each diagnosis overall (ASD, DS, i-DD, TD) and by sex [Female (F) or Males (M)].

**Dx**	**Sex**	**Class of metabolite**	**Pathway**	**Metabolite**	**Psychometric test**	**Subscale**	***P*^a^**	**r**
DS	F	Energy metabolites	Lipid metabolism	Carnitine	ABC	Lethargy/social withdrawal	0.011	−0.696
DS	Overall	Amino acids and derivatives	Lipid metabolism	Carnitine	ABC	Lethargy, social withdrawal	0.002	−0.64
DS	Overall	Others	Others	Myo inositol	VABS	Motor	0.011	−0.571
DS	Overall	Amino acids and derivatives	Lipid metabolism	O-Acetylcarnitine	MSEL	Receptive language	0.018	−0.555
DS	Overall	Energy metabolites	Glycolysis	Lactate	VABS	Communication	0.02	−0.551
i-DD	M	Amino acids and derivatives	Amino acid	Arginine	MSEL	Expressive language	0.039	−0.533
DS	Overall	Energy metabolites	Glycolysis	Lactate	VABS	Socialization	0.015	−0.532
DS	Overall	Amino acids and derivatives	Amino acid	Ornithine	ABC	Stereotypic behavior	0.035	−0.527
DS	Overall	Amino acids and derivatives	Lipid metabolism	O-Acetylcarnitine	MSEL	Composite score	0.019	−0.523
DS	Overall	Amino acids and derivatives	Amino acid	Creatine	VABS	Composite score	0.038	−0.522
DS	Overall	Energy metabolites	Glycolysis	Lactate	MSEL	Composite score	0.016	−0.512
DS	Overall	Amino acids and derivatives	Amino acid	Creatine	VABS	Socialization	0.025	−0.51
DS	Overall	Energy metabolites	Glycolysis	Lactate	VABS	Composite score	0.019	−0.489
DS	Overall	Amino acids and derivatives	Amino acid	Creatine	VABS	Motor	0.032	−0.478
DS	Overall	Energy metabolites	Glycolysis	Lactate	MSEL	Visual reception	0.021	−0.474
ASD	F	Amino acids and derivatives	Lipid metabolism	Carnitine	ABC	Lethargy/social withdrawal	0.042	−0.443
TD	F	Energy metabolites	Glycolysis	Pyruvate	VABS	Composite score	0.042	−0.381
ASD	M	Amino acids and derivatives	TCA	Fumarate	MSEL	Receptive language	0.037	−0.254
TD	M	Energy metabolites	Ketone bodies	3-Hydroxybutyrate	MSEL	Receptive language	0.046	−0.249
TD	Overall	Energy metabolites	Glycolysis	Pyruvate	VABS	Motor	0.007	−0.248
TD	M		Lipid metabolism	O-Acetylcarnitine	MSEL	Receptive language	0.048	−0.248
ASD	Overall	Energy metabolites	TCA	cis-Aconitate	MSEL	Visual reception	0.046	−0.211
ASD	Overall	Energy metabolites	TCA	cis-Aconitate	MSEL	Receptive language	0.032	−0.209
TD	Overall	Energy metabolites	Glycolysis	Pyruvate	MSEL	Receptive language	0.028	−0.207
TD	Overall	Energy metabolites	Glycolysis	Pyruvate	VABS	Daily living skills	0.023	−0.202
TD	Overall	Energy metabolites	Glycolysis	Pyruvate	MSEL	Fine motor	0.037	−0.185
TD	Overall	Energy metabolites	Glycolysis	Pyruvate	MSEL	Visual reception	0.034	−0.182
TD	Overall	Energy metabolites	Glycolysis	Pyruvate	VABS	Composite score	0.028	−0.181
TD	Overall	Energy metabolites	Glycolysis	Pyruvate	MSEL	Composite score	0.037	−0.171
ASD	Overall	Others	Others	Dimethyl sulfone	MSEL	Composite score	0.020	0.209
ASD	Overall	Others	Others	Dimethyl sulfone	MSEL	Expressive language	0.011	0.228
ASD	Overall	Others	Others	Dimethyl sulfone	MSEL	Receptive language	0.012	0.233
ASD	Overall	Others	Others	Dimethyl sulfone	VABS	Socialization	0.015	0.237
ASD	Overall	Others	Others	Dimethyl sulfone	VABS	Communication	0.024	0.242
ASD	M	Amino acids and derivatives	Amino acid	Glutamate	VABS	Socialization	0.023	0.285
i-DD	Overall	Others	Others	Creatinine (Crn)	MSEL	Composite score	0.025	0.365
i-DD	Overall	Others	Others	Creatinine (Crn)	VABS	Composite score	0.018	0.389
i-DD	Overall	Others	Others	Creatinine (Crn)	ABC	Lethargy, social withdrawal	0.032	0.393
i-DD	Overall	Others	Others	Creatinine (Crn)	VABS	Motor	0.022	0.393
i-DD	Overall	Others	Others	Creatinine (Crn)	MSEL	Visual reception	0.046	0.406
DS	Overall	Amino acids and derivatives	Amino acid	Arginine	ABC	Irritability, agitation, crying	0.026	0.508
DS	Overall	Amino acids and derivatives	Amino acid	Arginine	ABC	Hyperactivity, non-compliance	0.022	0.547
i-DD	M	Others	Others	Creatinine (Crn)	ABC	Lethargy/social withdrawal	0.022	0.558
i-DD	F	Others	Others	Dimethyl sulfone	ABC	Inappropriate speech	0.039	0.626
DS	M	Energy metabolites	Ketone bodies	Acetoacetate	MSEL	Fine motor	0.035	0.815
i-DD	F	Energy metabolites	BCAA and derivatives	3-Hydroxyisobutyrate (3-HIB)	ABC	Inappropriate speech	0.001	0.839

a *FDR adjusted p-values*.

*Only data showing significant associations p < 0.05 after FDR correction are presented. A coefficient r <|0.2| indicates essentially no correlation, |0.2 to <0.39| = small correlation, |0.4 to <0.59| = moderate correlation, |0.6 to <0.79| = high correlation, and r≥|0.8+| = very high correlation*.

Multiple linear regression (MLR) analysis was used to further examine the association between psychometric scores and plasma metabolites while adjusting for child's neurodevelopmental diagnosis, child's sex, child's age at blood draw, child's race/ethnicity, maternal age at child's birth, and parental homeownership (Supplementary Tables 2–4). MLR analysis demonstrated significant associations between 19 plasma metabolites and psychometric scores−9 of which (glycolysis metabolites (lactate and pyruvate), ketone bodies (3-hydroxybutyrate and acetoacetate), TCA cycle metabolites (cis-aconitate and fumarate), ornithine, and dimethyl sulfone) overlapped with metabolites identified by Spearman correlation (Supplementary Table 5).

Negative associations were seen for 14 plasma metabolites indicating poorer scores were associated with elevated plasma metabolite levels based on MLR analysis.

- On the MSEL, reduced visual reception correlated with elevated lactate (β = −10.03, *p* = 0.035), and cis-aconitate (β = −13.53, *p* = 0.007). Poorer receptive language was associated with elevated ornithine (β = −17.86, *p* = 0.012), tryptophan (β = −9.35, *p* = 0.015), lactate (β = −10.40, *p* = 0.038), pyruvate (β = −7.70, *p* = 0.022), o-acetylcarnitine (β = −17.18, *p* = 0.002), cis-aconitate (β = −14.00, *p* = 0.008), fumarate (β =-13.62, *p* = 0.028), and succinate (β = −15.83, *p* = 0.008). Poorer expressive language correlated with elevated formate (β = −16.54, *p* = 0.036), and glycolate (β = −19.07, *p* = 0.016). Finally, poorer MSEL composite score (combining over all of the subscales) correlated with elevated cis-aconitate (β = −8.91, *p* = 0.032), fumarate (β = −10.08, *p* = 0.038), and succinate (β = −10.32, *p* = 0.028).
- On the VABS, poorer communication skills correlated with elevated plasma ornithine (β = −14.01, *p* = 0.040).Additionally, poorer daily living skills correlated with elevated lactate (β = −9.32, *p* = 0.027), and succinate (β = −10.03, *p* = 0.048). Reduced socialization skills correlated with higher plasma N,N-Dimethylglycine (β = −13.16, *p* = 0.029), ornithine (β = −21.33, *p* = 0.002), and lactate (β = −10.14, *p* = 0.036). Reduced motor skills correlated with elevated glutamine (β = −13.27, *p* = 0.023), ornithine (β = −13.59, *p* = 0.042), and acetone (β = −12.29, *p* = 0.037). Overall, poorer VABS composite score correlated with elevated ornithine (β = −13.83, *p* = 0.009), and lactate (β = −8.93, *p* = 0.017).
- On the ABC, increased irritability, agitation and crying correlated with elevated hypoxanthine (β = −1.53, *p* = 0.034).Additionally, increased lethargy/social withdrawal correlated with elevated o-acetylcarnitine (β = −2.55, *p* = 0.030), and ornithine (β = −3.18, *p* = 0.025).

Positive associations were also observed for five metabolites based on MLR analysis suggesting improved scores were associated with higher levels of certain plasma metabolites.

- On the MSEL, plasma dimethyl sulfone positively correlated with improvements in composite MSEL score (β = 8.30, *p* = 0.027), MSEL receptive language (β = 11.39, *p* = 0.017), and MSEL expressive language (β = 9.88, *p* = 0.030).
- On the VABS, improved daily livings skills correlated with higher plasma isoleucine (β = 11.56, *p* = 0.048), valine (β =15.15, *p* = 0.036), and 3-hydroxybutyrte (β = 5.13, *p* = 0.027).
- Positive associations were also seen with ABC subscales where reduced stereotypic behavior correlated with higher plasma acetoacetate (β = 0.59, *p* = 0.024).

## Discussion

The results of this study document significant correlations between several plasma metabolites and cognitive skills, adaptive function, and aberrant/maladaptive behavior in CHARGE study participants with DS, i-DD, ASD, and TD. Most of these associations differed across the diagnostic groups while some were sex- and diagnosis-specific—and will be the focus of this discussion. Nonetheless, even after adjusting for the child's neurodevelopmental diagnosis, sex, and other possible confounders, the correlations between metabolites and psychometric scores were preserved.

**Figure 6:**
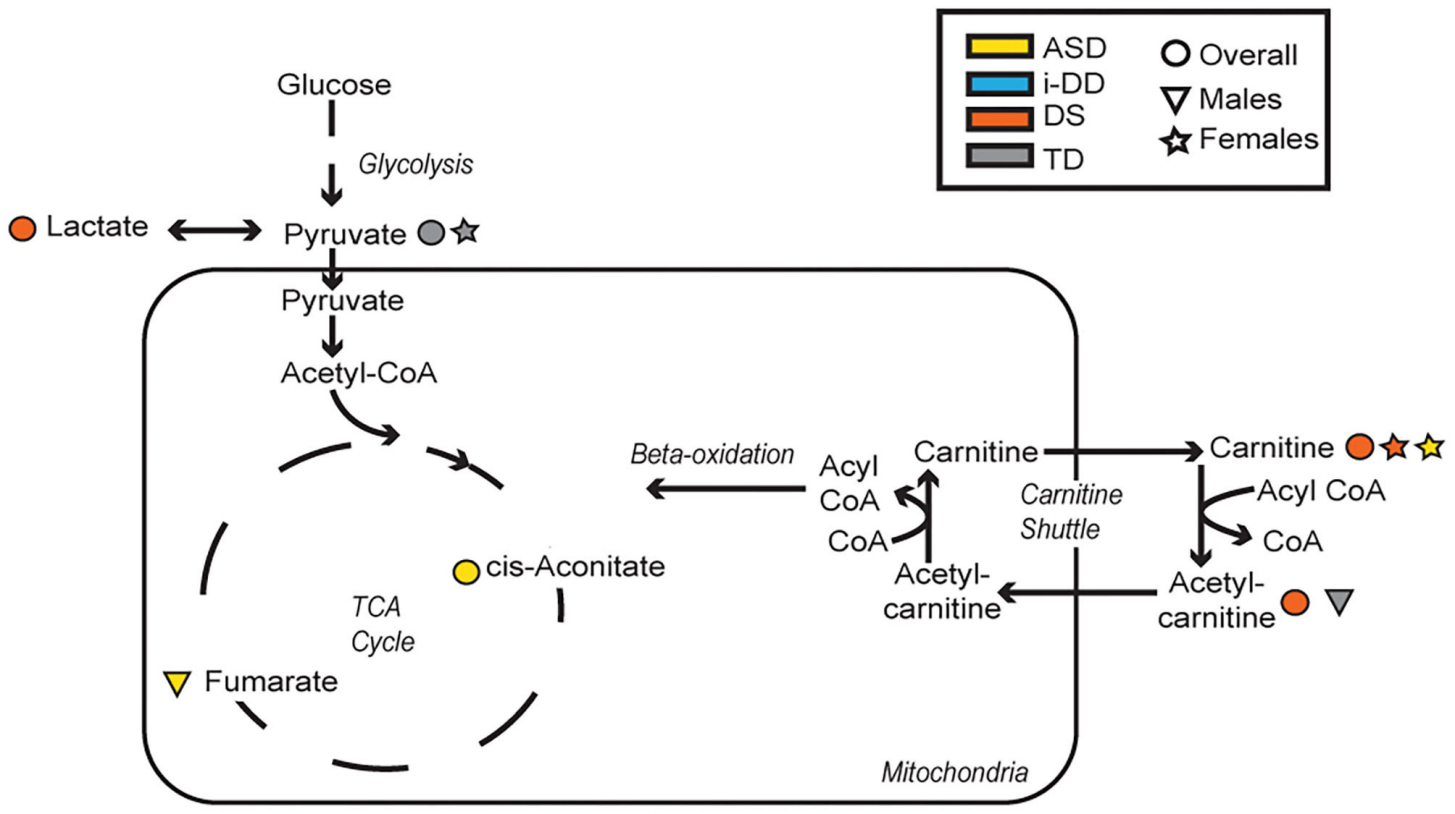
Metabolic pathways converging glycolysis, tricarboxylic acid cycle (TCA) cycle, and carnitine shuttle. Significant associations between plasma metabolites and psychometric scores are presented with different shapes representing if the association (positive or negative) was seen overall or in a sex-specific manner (circle=Overall, triangle=Males, star=Females) and in which group (yellow=ASD, blue=i-DD, orange=DS, gray=TD).

In general, we found that elevated levels of metabolites involved in cellular energy and mitochondrial function correlated with deficits in psychometric scores (Figure 6). Elevated TCA cycle intermediates (fumarate and cis-aconitate) correlated with poorer scores among ASD cases. Higher concentrations of cis-aconitate correlated with poorer visual reception, and receptive language on the MSEL among ASD cases overall. Although we did not specifically test the significant difference between males and females, the trend of this association appears to be more pronounced among males with ASD, as elevated fumarate also associated with deficits in receptive language. The TCA cycle takes place in the mitochondrial matrix and is critical for many cellular processes. Mitochondrial dysfunction has been reported to affect ~5% of individuals with ASD (35). Mitochondrial DNA is inherited uniparentally through the maternal germline, contrastingly to all nuclear genes which are built from a combination of father's and mother's genetic material (36). Some have suggested this maternal inheritance of mitochondrial DNA may have substantial sex-specific consequences resulting in reduced evolutionary male fitness (37). Interestingly, certain developmental delays, language and speech pathologies, dyslexia, and autism predominantly affect boys (38–40), though it is uncertain if/how mitochondrial DNA may play a role in these disorders. Elevated plasma lactate and pyruvate (products of glycolysis), which serve as peripheral markers of metabolism dysfunction, were found to be associated with decreased psychometric scores. Specifically, we found that among DS cases, elevated lactate correlated with poorer visual reception and overall MSEL scores, as well as with poorer communication, socialization, and overall VABS scores. Additionally, among TD cases, elevated pyruvate, also correlated with deficits in fine motor, receptive language, visual reception, overall MSEL score, as well as poorer daily living skills, motor skills, and poorer overall adaptive behavior on the VABS. Interestingly, the association between elevated pyruvate and deficits in overall VABS scores was particularly strong among TD females. Elevated lactate and pyruvate concentrations have previously been identified among children with DS (20) and children with ASD (41). Even after adjusting for the child's neurodevelopmental diagnosis, child's sex, and other potential confounders in MLR models, elevated levels of these mitochondrial related metabolites strongly correlated with deficits in multiple domains of cognitive function, and adaptive skills. Elevated plasma lactate strongly correlated with poorer visual reception and receptive language on the MSEL, and poorer daily living skills, socialization skills, and overall deficits in VABS scores. Elevated plasma TCA cycle intermediates (cis-aconitate, fumarate, and succinate) correlated with deficits in overall MSEL score and receptive language. Elevated plasma cis-aconitate also correlated with deficits in visual reception on the MSEL and elevated succinate correlated with poorer daily living skills on the VABS.

Additionally, elevated plasma o-acetylcarnitine correlated with deficits in receptive language and poorer composite MSEL scores among DS cases, as well as a poorer receptive language among TD boys. Interestingly, girls with ASD and DS share altered carnitine in association with increased maladaptive behavior (increased lethargy/social withdrawal on the ABC). Carnitine and acetylcarnitine help facilitate the transport of activated fatty acids into the mitochondria for lipid metabolism. As such, these results suggest that dysregulated carnitine/acetylcarnitine lipid metabolism may negatively impact cognitive score domains and maladaptive behavior. Moreover, carnitine also has a neuroprotective role, guarding the lipid membranes against oxidative damage. Oxidative damage to lipid membranes may play a role in the Alzheimer-type symptoms common in DS. Interestingly, Alzheimer's Disease disproportionately affects more women than men in the general population (42) and specifically, more women than men with DS (43). These metabolic deficiencies of lipid metabolism, although speculative, could be partly responsible for the sex differences in both DS and Alzheimer's, and may provide a mechanism to explain aspects of both development and degeneration. However, future longitudinal research would be required to further investigate this, given the ages of our study population. Collectively, these findings suggest that dysregulated mitochondrial and cellular energy production negatively impacts cognitive/behavioral scores. However, ASD is particularly impacted by elevated TCA cycle metabolites, while alterations in lipid metabolism and peripheral mitochondrial metabolites predominantly affect DS cases and TD controls.

Some diagnosis-specific associations between plasma metabolites and psychometric scores were also found. Among DS cases, we found that elevated myo-inositol concentrations correlated with poorer motor skills on the VABS among DS cases. Myo-inositol can be obtained from the diet or endogenously produced. It plays an important role as a second messenger (44) and in the composition of phospholipids (45). Although DS cases tended to have higher myo-inositol levels compared to TD controls, this was not significantly different (Supplementary Table 1). However, increased levels of myo-inositol have previously been reported in patients with complex regional pain syndrome, a neurological disorder (46). Elevated myo-inositol has also been reported (47) in elderly patients with Alzheimer's disease (48), suggesting abnormalities in the inositol messenger pathway occur early in the etiology of Alzheimer's. Conversely, among boys with ASD, we found a weak positive association between elevated glutamate and improved socialization score on the VABS adaptive behavior. Glutamate is a major excitatory neurotransmitter. It is highly concentrated throughout the brain and is critical to neuronal plasticity and the maintenance of cognitive functioning (49), though here we found that it correlated with higher adaptive skills among ASD cases.

Despite no known genetic diagnosis, children with i-DD also showed correlations between plasma metabolites and psychometric measurements—surprisingly, most were positive associations (i.e., higher metabolite concentrations correlated with improved scores). For example, among i-DD cases overall, elevated creatinine (Crn) levels correlated with higher visual reception, composite MSEL score, motor skills, overall better VABS score, as well as decreased lethargy/social withdrawal on the ABC. Among boys with i-DD, elevated plasma Crn more strongly correlated with better functioning in lethargy/social withdrawal on the ABC. Crn is a breakdown product of creatine phosphate in muscle and is usually produced at a fairly constant rate in the body. However, when kidney function is impaired, Crn levels rise. Although the level of Crn among our i-DD cases still falls within normal ranges (metabolite concentrations presented in Supplementary Table 1) (50), it is intriguing that elevated levels of Crn had positive associations with psychometric scores among i-DD cases. Given that males generally have greater Crn production than females (51), this may explain why these associations were more prominent among-DD males.

Among girls with i-DD, plasma 3-hydroxyisobutyrate (3-HIB) positively correlated with reduced inappropriate speech on the ABC. However, elevated levels of 3-HIB have previously been associated with insulin resistance in animal models (52) and future risk of type-2-diabetes among obese individuals (BMI > 30) (53). 3-HIB is a catabolite of the essential branched-chain amino acid (BCAA) valine and serves as a reporter of BCAA utilization. BCAAs have important mediation effects on protein synthesis, glucose homeostasis, and fatty acid utilization (54). Moreover, gender differences have been reported in the regulation of BCAA catabolism, potentially due to sex-hormones (55). Our results suggest 3-HIB may signify increased BCAA utilization among i-DD females which has positive effects on behavior in the short-term, but long-term effects (such as future insulin resistance) remain unknown.

Additionally, elevated plasma dimethyl sulfone also correlated with reduced inappropriate speech among i-DD females. Similarly, among ASD cases, dimethyl sulfone also correlated with improved expressive language, receptive language, and composite MSEL score, as well as higher communication skills and socialization skills on the VABS. Dimethyl sulfone can be derived from various sources including diet, human endogenous methanethiol metabolism, and intestinal microbiota metabolism (56). Dimethyl sulfone can also readily transfer across the blood-brain barrier and has previously been identified in cerebrospinal fluid, although the neurological consequences of it are uncertain. Interestingly, dimethyl sulfone has gained anecdotal repute for the treatment of pain and inflammatory conditions (57), but there is little published scientific research to support its use (58), and there is currently no Recommended Dietary Allowance (RDA) for dimethyl sulfone. Although collectively these observations on dimethyl sulfone seem intriguing, further investigations into the effects of dimethyl sulfone, particularly in vulnerable populations such as those with developmental delays, are necessary.

Mixed results were seen with plasma ketone bodies. Among boys with DS elevated acetoacetate strongly correlated with improved fine motor skills on the MSEL. However, elevated plasma 3-hydroxybutyrate (another ketone body) was associated with slightly lower levels of receptive language on the MSEL among TD males. Ketone bodies are produced by the liver and used peripherally as an energy source when glucose is not readily available providing the brain with an alternate source of energy. There is some evidence that ketone bodies may be neuroprotective through possible mechanisms of anti-oxidative stress, and maintaining energy supply (57). DS represents one of the most well-documented cases related to redox imbalance partly attributed to overexpression of superoxide dismutase (SOD-1) encoded by chromosome 21 (59). Therefore, acetoacetate may be eliciting beneficial effects in DS as an antioxidant. Alternatively, ketone utilization may simply differ between DS males and TD males.

**Figure 7:**
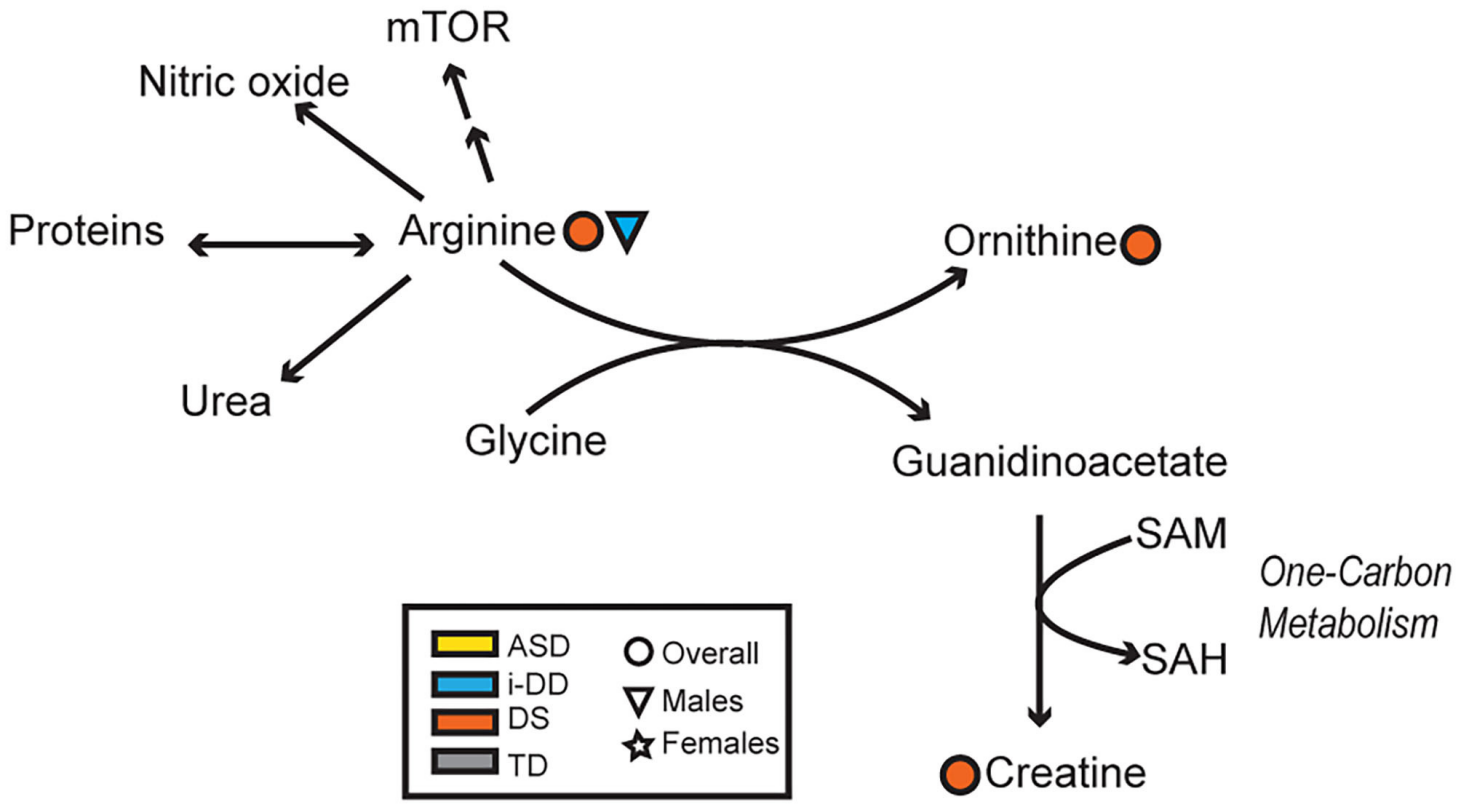
Overview of the arginine-creatine pathway and arginine metabolism. Significant associations between plasma metabolites and psychometric scores are presented with different shapes representing if the association (positive or negative) was seen overall or in a sex-specific manner (circle=Overall, triangle=Males, star=Females) and in which group (yellow=ASD, blue=i-DD, orange=DS, gray=TD). (mTOR, Mammalian Target of Rapamycin; SAM, S-adenosylmethionine; SAH, S-adenosylhomocysteine).

Mixed results were also seen concerning the amino acid arginine. Elevated arginine was associated with reduced hyperactivity/non-compliance in the ABC among DS cases, but deficits in expressive language on the MSEL among i-DD boys. Arginine plays important functions in numerous metabolic pathways including protein synthesis, the formation of nitric oxide (NOS), and it is an inducer of the Mammalian Target of Rapamycin (mTOR) pathway. Interestingly, several studies have found that upregulation in the Akt/mTOR pathway, which regulates translation at dendritic spines was associated with idiopathic ASD (60, 61) and was also found to be correlated with social deficits in animal models (62). Similarly, the upregulated mTOR pathway has also been postulated in DS and may be a factor in the accumulation of cellular amyloid-β proteins contributing to the pathogenesis of Alzheimer's disease among DS cases (63, 64). Indeed mTOR is implicated in a variety of biological pathways and is pivotal for proper brain development (65), which may provide clues as to how arginine may affect cognition, language, and behavior. Yet, among DS cases, arginine also may be related to alterations in the arginine-creatine pathway (a pathway negatively impacted among patients with kidney transplants)—of relevance considering kidney disease is a frequent complication in DS (66). Arginine can be used to synthesize creatine, but this is dependent on a methyl-group donor from S-adenosylmethionine (Figure 7). We have previously shown dysregulated one-carbon metabolism among DS cases (17). Moreover, we found that by-products of this cycle correlated with poorer psychometric scores in DS cases. Moreover, elevated plasma creatine levels in the DS sample correlated with deficits in motor skills, socialization, and composite VABS score, and elevated ornithine correlated with increased stereotypic behavior as measured on the ABC. Collectively, this may suggest that in DS, the metabolic flux of arginine may impact behavior differently.

Although the discussed metabolites highlight differences based on neurodevelopmental diagnosis, and sex-differences, many of these associations were preserved even after controlling for child's diagnosis, child's sex, child's race/ethnicity, child's age at blood draw, maternal age at child's birth and socioeconomic factors such as parental homeownership, as shown in MLR analysis. Specifically, these key metabolites included glycolysis metabolites (lactate and pyruvate), ketone bodies (3-hydroxybutyrate and acetoacetate), TCA cycle metabolites (cis-aconitate and fumarate), as well as ornithine—which were associated with deficits in multiple domains of cognitive function, adaptive skills, and deviant behaviors, including numerous subscales, supporting a global dysregulation of neurodevelopment –and dimethyl sulfone. Additionally, the direction of these associations agreed between spearman correlation analysis and MLR analysis. Except for dimethyl sulfone (which was positively correlated with psychometric scores), elevated levels of these metabolites correlated with poorer psychometric scores.

Yet, despite strong correlations observed among plasma metabolites and psychometric scores, particularly in the DS and i-DD children, there are several limitations in the present study. First, plasma metabolites were measured after a neurodevelopmental diagnosis was made. As such, it is not possible to conclude whether differences in these metabolic compounds contribute to the onset of any of these conditions or their symptoms, particularly for ASD and i-DD cases. Yet, some of the behavioral correlations observed in DS cases would seem almost certainly a result of the gene products on chromosome 21 causing biochemical changes. Additionally, because we performed a secondary analysis of samples already collected in an epidemiological study, samples were also not fasted before collection, and the storage duration of these samples varied considerably as the CHARGE study began specimen collections in 2004. As such, this variability in storage time may have affected the quality of plasma samples. However, these factors would have equally affected the TD samples as compared with any of the diagnoses, and therefore the associations observed are unlikely to be chance findings. Although demographics in our study population were balanced across cases and controls, we did not adjust our findings for some sociodemographic factors which might influence a child's performance, for example, parental education, mother's birthplace. However, the MLR results did adjust for other SES variables that are strongly correlated with these: maternal age, race/ethnicity, and homeownership.

This study is unique in that we were able to utilize the existing case-control epidemiologic CHARGE study to investigate the plasma metabolome and leverage the extensive infrastructure of meta-data available. CHARGE has contributed substantially to the current knowledge of the environmental components relating to autism (17, 67–69). Strengths in our study included the fact that both controls and cases were recruited based on the same eligibility criteria, and that ASD and developmental delay diagnoses were clinically confirmed (as was TD) by trained psychometricians who demonstrated reliability on the tests they administered. All the instruments used for the child assessments are standardized and normed and widely used, for the ages of the CHARGE study population, thereby enabling us to examine the metabolic profiles with well-characterized behavioral and developmental phenotypes based on specific functional domains in both children with developmental disabilities and controls selected from the same population. We are not aware of any study of this size that evaluated a wide range of metabolites in relation to these diagnoses. A further strength was that the children were all within a relatively narrow age range, and finally, we also were able to investigate and identify sex differences, even though the sample size for girls was limited.

Our results highlight the potential importance of altered metabolism in children with developmental disabilities to explaining variation in the severity of deficits in cognitive and adaptive abilities and behavioral phenotypes. These results could help guide potential metabolic intervention strategies (such as targeted mitochondrial function, TCA cycle metabolism, or arginine metabolism) to improve symptoms in children with developmental delays, even among those with a known genetic origin.

## Conclusion

We identified several correlations between psychometric and metabolic phenotypes among children with three different diagnostic categories of developmental disabilities—ASD, DS, and i-DD—as well as the broader population of typically developing controls. Several behavioral/ neurodevelopmental instruments (ABC, MSEL, and VABS) enabled us to examine the associations between metabolites from key biochemical pathways and specific functional subdomains within cognitive, adaptive, and behavioral development. Although some overlap in findings was seen across a child's diagnosis, most were specific to the diagnosis (such as the correlation of elevated TCA cycles metabolites negatively affecting MSEL scores among ASD cases). Moreover, some sex-specific differences were observed across diagnoses, e.g., elevated carnitine similarly correlated with increased lethargy and social withdrawal on the ABC among girls with DS and ASD.

## Data Availability Statement

The raw data supporting the conclusions of this article will be made available by the authors, without undue reservation.

## Ethics Statement

The studies involving human participants were reviewed and approved by State of California Department of Developmental Services and the institutional review boards at the University of California, Davis, and Los Angeles. Written informed consent to participate in this study was provided by the participants' legal guardian/next of kin.

## Author Contributions

JS-O processed and analyzed the metabolomics data and took the lead in writing the manuscript. All authors provided critical feedback and helped shape the research and analysis of the manuscript.

## Conflict of Interest

The authors declare that the research was conducted in the absence of any commercial or financial relationships that could be construed as a potential conflict of interest.
